# Is atrial fibrillation in HFpEF a distinct phenotype? Insights from multiparametric MRI and circulating biomarkers

**DOI:** 10.1186/s12872-024-03734-0

**Published:** 2024-02-07

**Authors:** Abhishek Dattani, Emer M. Brady, Prathap Kanagala, Svetlana Stoma, Kelly S. Parke, Anna-Marie Marsh, Anvesha Singh, Jayanth R. Arnold, Alastair J. Moss, Lei Zhao, Mary Ellen Cvijic, Matthew Fronheiser, Shuyan Du, Philippe Costet, Peter Schafer, Leon Carayannopoulos, Ching-Pin Chang, David Gordon, Francisco Ramirez-Valle, Michael Jerosch-Herold, Christopher P. Nelson, Iain B. Squire, Leong L. Ng, Gaurav S. Gulsin, Gerry P. McCann

**Affiliations:** 1 Department of Cardiovascular Sciences, University of Leicester and the National Institute for Health Research Leicester Biomedical Research Centre, Glenfield Hospital, Leicester, UK; 2 University of Liverpool, Liverpool, UK; 3 Bristol Myers Squibb, Princeton, NJ USA; 4 Brigham and Women’s Hospital and Harvard Medical School, Boston, USA

**Keywords:** Heart failure, Atrial fibrillation, Cardiovascular magnetic resonance, Biomarkers, Phenotype, Cluster analysis

## Abstract

**Background:**

Heart failure with preserved ejection fraction (HFpEF) and atrial fibrillation (AF) frequently co-exist. There is a limited understanding on whether this coexistence is associated with distinct alterations in myocardial remodelling and mechanics. We aimed to determine if patients with atrial fibrillation (AF) and heart failure with preserved ejection fraction (HFpEF) represent a distinct phenotype.

**Methods:**

In this secondary analysis of adults with HFpEF (NCT03050593), participants were comprehensively phenotyped with stress cardiac MRI, echocardiography and plasma fibroinflammatory biomarkers, and were followed for the composite endpoint (HF hospitalisation or death) at a median of 8.5 years. Those with AF were compared to sinus rhythm (SR) and unsupervised cluster analysis was performed to explore possible phenotypes.

**Results:**

136 subjects were included (SR = 75, AF = 61). The AF group was older (76 ± 8 vs. 70 ± 10 years) with less diabetes (36% vs. 61%) compared to the SR group and had higher left atrial (LA) volumes (61 ± 30 vs. 39 ± 15 mL/m^2^, *p* < 0.001), lower LA ejection fraction (EF) (31 ± 15 vs. 51 ± 12%, p < 0.001), worse left ventricular (LV) systolic function (LVEF 63 ± 8 vs. 68 ± 8%, *p* = 0.002; global longitudinal strain 13.6 ± 2.9 vs. 14.7 ± 2.4%, *p* = 0.003) but higher LV peak early diastolic strain rates (0.73 ± 0.28 vs. 0.53 ± 0.17 1/s, *p* < 0.001). The AF group had higher levels of syndecan-1, matrix metalloproteinase-2, proBNP, angiopoietin-2 and pentraxin-3, but lower level of interleukin-8. No difference in clinical outcomes was observed between the groups. Three distinct clusters were identified with the poorest outcomes (Log-rank *p* = 0.029) in cluster 2 (hypertensive and fibroinflammatory) which had equal representation of SR and AF.

**Conclusions:**

Presence of AF in HFpEF is associated with cardiac structural and functional changes together with altered expression of several fibro-inflammatory biomarkers. Distinct phenotypes exist in HFpEF which may have differing clinical outcomes.

**Supplementary Information:**

The online version contains supplementary material available at 10.1186/s12872-024-03734-0.

## Introduction

Heart failure with preserved ejection fraction (HFpEF) remains a well-recognised therapeutic challenge which may be due to the vast heterogeneity of this complex systemic syndrome. HFpEF may be associated with the presence of distinct disease phenotypes, which involve divergent cellular and molecular based pathophysiological mechanisms [1, 2]. Many (up to two thirds) but not all patients with HFpEF develop atrial fibrillation (AF) [3] and AF is a major component of diagnostic scoring systems for HFpEF [4]. There may be differences in the types of HFpEF patients who develop AF [1] and some subtypes of HFpEF appear to have lower AF prevalence [5]. The presence of AF in HFpEF may be associated with worse clinical outcomes [6, 7].

The variable penetrance of AF in HFpEF remains incompletely understood and mechanisms behind the coexistence of these conditions has not been fully elucidated. It is posited that AF in HFpEF occurs as a consequence of a unified atrial and ventricular myopathy that could be explained by systemic inflammation and fibrosis [8–10].

Cardiovascular magnetic resonance (CMR) imaging has become the reference standard for the assessment of left atrial (LA) volumes and left ventricular (LV) volumes, mass and systolic function assessment [11]. Additionally, use of gadolinium allows the detection of ischaemia and diffuse and focal fibrosis [12], and tissue tracking enables quantitation of myocardial strain and strain rates for a unique evaluation of HFpEF [13]. Whilst previous studies have described the role of AF in HFpEF, there remain limited studies utilising CMR to characterise patients with HFpEF and concurrent AF particularly in the assessment of CMR-derived markers of diastolic dysfunction and in combination with circulating biomarkers [8, 14, 15]. There are no studies that have used such extensive multidimensional assessment in a single group of HFpEF patients to explore the possibility of AF representing a distinct phenotype.

The DIAMOND-HFpEF study recruited participants from 2013 to 2015 and aimed to better phenotype and characterise patients with HFpEF and provide mechanistic insights into pathophysiology. Our previous analyses have demonstrated the prognostic significance of focal and diffuse fibrosis [16], microvascular dysfunction [12] and right ventricular dysfunction in HFpEF [17]. In this secondary analysis, we aimed to comprehensively characterise this ethnically diverse group of HFpEF patients with and without AF using multidimensional CMR and circulating plasma fibroinflammatory biomarkers and apply the unsupervised machine learning technique of *k*-means to determine if phenotypic sub-groups (clusters) within this HFpEF cohort could be identified. We hypothesised that AF represents a distinct phenotype in HFpEF.

## Methods

### Study design and participants

This was a secondary analysis of the DIAMOND-HFpEF study (NCT03050593) in which adults with clinical or radiographic evidence of HF and an LV EF ≥ 50% on transthoracic echocardiography (TTE) were prospectively recruited. Key exclusion criteria were: myocardial infarction in the previous six months, possible diagnosis of cardiomyopathy or constrictive pericarditis, severe valve disease or pulmonary disease (predicted forced expiratory volume in one second of < 30% or a predicted forced vital capacity of < 50%), estimated glomerular filtration rate (eGFR) < 30 ml/min/1.73m^2^ and contraindication to CMR. The study complies with the Declaration of Helsinki, the study was approved by the East Midlands Research Ethics Committee (12/EM/0222), and all participants provided written informed consent to participate in the study.

### Assessments

Participants underwent baseline clinical assessment including medical history, 12-lead electrocardiogram (ECG), blood sampling and functional assessment, prior to TTE and CMR. Participants were placed in the AF group if they had a known baseline diagnosis of permanent, persistent or paroxysmal AF as described by the patient history or medical notes, or if incidental AF was detected on the 12-lead ECG or during CMR. Participants who had no known history of AF and were shown to have sinus rhythm (SR) on their 12-lead ECG and during their CMR were placed in the SR group.

### Blood sampling

Blood samples were taken at the time of recruitment and included renal function and haematocrit. Circulating biomarkers were quantified by a bead-based multiplex assay on a Luminex platform (Bristol Myers Squibb, NJ, USA) as previously described [18]. The panel included 49 biomarkers associated with fibrosis, myocardial injury, atrial stretch, myocardial hypertrophy, inflammation and oxidative stress, renal markers and endothelial dysfunction (Supplementary Table S1).

### Functional assessment

Functional assessments were undertaken using the New York Heart Association (NYHA) functional class, Minnesota Living with Heart Failure Questionnaire [19] and standardised six-minute walk test (6MWT).

### TTE

Echocardiography was performed and reported by one of two accredited sonographers using an iE33 system with an S5–1 transducer (Philips Medical Systems, Best, The Netherlands). Image acquisition and reporting was undertaken as per American Society of Echocardiography guidelines [20]. Doppler measurements were averaged over five cardiac cycles for participants in AF.

### CMR imaging

Subjects underwent adenosine-stress CMR imaging using a 3-Tesla scanner (Siemens Skyra, Erlangen, Germany) with an 18-channel cardiac coil. Detailed imaging acquisition protocols have been previously described [16]. Briefly, both long- and short-axis cine imaging were undertaken using balanced steady-state free precession technique with coverage of the whole heart. Retrospective ECG gating was used for those in SR and prospective gating for those with AF or frequent ectopics. At mid-LV level, T1 maps were acquired pre- and post-contrast using a modified inversion recovery Look-Locker technique [21]. Perfusion imaging was performed at rest and during pharmacological stress using 140–210 μg/kg/min of adenosine infused for 3–5 minutes. Stress and rest perfusion images were obtained at basal, mid-ventricular and apical levels following the administration of 0.04 mmol/kg of a gadolinium-based contrast agent (Gadovist, Bayer Healthcare) as previously reported [12]. Late gadolinium enhancement (LGE) imaging at the same slice positions as cine imaging was performed using a T1-weighted segmented inversion-recovery gradient echo sequence 10–15 minutes following a top-up dose of 0.07 mmol/kg of contrast (total contrast dose of 0.15 mmol/kg).

### CMR analysis

All images were batch analysed offline for structure and function analysis by a single observer, blinded to participant details, using Circle CVI software (Circle Cardiovascular Imaging, CVI42 v5.10.1, Calgary, Canada). For LV volume and mass measurement, the built-in automated contouring tool was used on the short-axis stack with adjustments only made for clear and obvious errors. Participants with a CMR-derived LV EF of < 45% were excluded from further analysis. Biplane LA volumes were calculated using the 4- and 2-chamber cine images using the automated tool to contour throughout the cardiac cycle to generate a maximum and minimum LA volume in addition to LA EF.

Myocardial strain measurement was performed using tissue tracking as previously described [22] to calculate global longitudinal strain (GLS) and global circumferential strain (GCS) as well as longitudinal and circumferential peak early diastolic strain rate (PEDSR). Strain values are presented as absolute values such that lower values indicate worse myocardial mechanics [23]. Pre- and post-contrast T1 maps were used to calculate myocardial extracellular volume fraction using haematocrit sampled on the same day of the CMR scan [24]. Given the differences in temporal resolution between retrospective and prospective imaging, ten scans in each group underwent repeat analysis for intra-observer variability.

Perfusion analysis was undertaken by two experienced observers. Participants with perfusion defects in a coronary territory distribution or with an infarct were excluded from quantitative analysis. This was performed by generating endocardial and epicardial contours (MASS, Medis Medical Imaging Systems, Leiden, The Netherlands) using model-independent deconvolution of myocardial signal intensity curves which generated absolute myocardial blood flow in mL/min/g as previously described [12]. LGE images were analysed qualitatively by two experienced observers and graded as present or absent with further characterisation into myocardial infarction or non-ischaemic patterns of fibrosis.

### Clinical follow up

An exploratory analysis of outcomes was performed as part of this analysis. The clinical endpoint was a composite of HF hospitalisation (defined as the primary reason for admission to hospital requiring treatment with either intravenous diuretic, renal dose dopamine or intravenous nitrate medication) or all-cause mortality. Electronic hospital records and patient clinical notes were sourced to obtain outcome data by a single observer who was blinded to AF status. Participants were followed up until the first event occurred or up to the time of data capture (right censoring). Follow up time was determined as the time from study enrolment up to the first event or censorship, and was measured in days and converted to months/years as appropriate.

### Statistical analysis

All data were assessed for outliers and removed if appropriate prior to analysis. Continuous variables underwent normality testing using histograms, Q-Q plots and the Shapiro-Wilk normality test. Normally distributed variables are presented as mean +/− standard deviation and non-normally distributed variables are presented as median (interquartile range). Baseline characteristics were compared using an independent T-test, Mann-Whitney U test or Chi-squared test as appropriate. Comparison of imaging data were adjusted for important clinical characteristics that are known to affect cardiac structure and function (including age, sex, ethnicity, body mass index (BMI), eGFR, systolic blood pressure (SBP) and diabetes status) using ANCOVA. Depending on data distribution and percentage of plasma biomarkers at the lower limit of detection, they were Log10 transformed or dichotomise as described in Supplemental Materials. Biomarkers were then compared between groups using ANCOVA with the same covariates as for imaging data. Given the heterogeneity of AF, a sensitivity analysis excluding participants with paroxysmal AF was also performed.

#### Cluster analysis

To determine if AF in HFpEF represented a distinct phenotype, we employed unsupervised K-means clustering [25]. Multivariate Imputation by Chained Equations [26] was used for handling missing data with 10 imputations conducted. Outliers were removed, data scaled and K-means clustering was performed for 2 to 8 clusters with feature selection and Pearson correlation, as the clustering distance measure, on all 10 datasets. A total of 63 variables were selected for cluster analysis (Supplemental Table S3) and all used for feature selection (Feature importance in K-means clustering [27]), selection based on misclassification threshold > 0.02 and counted ≥2/imputed dataset, for K-3. The data were then pooled and participants coded by cluster assignment to permit reporting descriptive statistics within each cluster. Generalised linear modelling was used to determine if cluster assignment was predictive of AF status. The demographics and key clinical variables were compared between the three clusters (continuous data with Wilcoxon rank sum test, categorical data with Fisher’s exact test for proportions (2 groups) and ANOVA (3 groups)).

#### Patient outcome data

Kaplan-Meier survival curves to the composite endpoint of hospitalisation for heart failure and all-cause death were generated stratified by AF status initially and then by cluster assignment. The Log-rank test was used to assess differences in outcomes between groups and clusters, with time-to-first event used in cases of multiple events of heart failure hospitalisation. Hazard ratios for impact of AF were generated using Cox regression analysis with key clinical outcomes as covariates (age, eGFR and the presence of hypertension, diabetes, chronic obstructive pulmonary disease and previous HF hospitalisation).

For all analyses, *P*-value < 0.05 was considered statistically significant. Descriptive statistics, ANCOVA and survival curves were performed using SPSS Statistics (version 28.0, IBM Corp., Armonk, New York, USA). K-means cluster analysis was conducted in RStudio (RStudio Team (2020); http://www.rstudio.com/)*.* Graphs were generated using SPSS Statistics and GraphPad Prism (version 9.0.0, San Diego, California, USA).

## Results

### Baseline characteristics

**Fig. 1:**
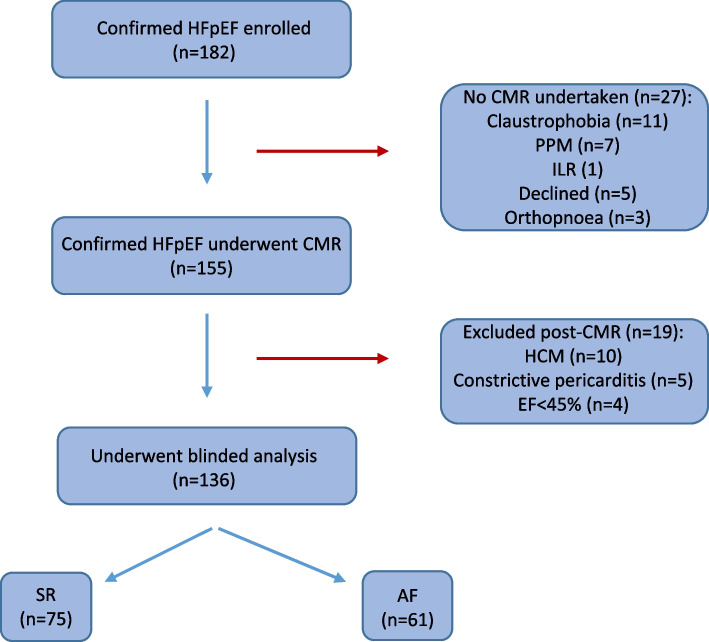
Study overview showing study population and reasons for exclusion. Abbreviations: PPM = permanent pacemaker; ILR = implantable loop recorder; HCM = hypertrophic cardiomyopathy

182 participants with HFpEF enrolled into the study with 136 included in these analyses, and stratified by AF status: SR (*n* = 75) and AF (*n* = 61). Study recruitment and reasons for exclusion are shown in Fig. 1.

Participant baseline characteristics are presented in Table 1. Compared to the SR group, on average the AF group was older, had a lower prevalence of diabetes and was more likely to be of White ethnicity. Sex distribution, body mass index and other comorbidities were similarly distributed between groups. No difference in subjective or objective functional status (NYHA class, 6MWT and the Minnesota Living with Heart Failure Questionnaire scores) was evident between groups. The AF group had higher BNP, proBNP and N-terminal proatrial natriuretic peptide (NT-proANP) levels. A higher proportion of patients in the AF group were taking beta blockers and digoxin.

**Table 1:** Baseline characteristics comparing HFpEF participants with and without AF

	Sinus rhythm (*n* = 75)	AF (*n* = 61)	*P* value
Age, years	70 ± 10	76 ± 8	**< 0.001**
Sex, n (%) female	39 (52)	30 (49)	0.744
Ethnicity			**0.023**
White, n(%)	57 (76)	57 (93)	
South Asian, n(%)	14 (19)	3 (5)	
Black and Other, n(%)	4 (5)	1 (2)	
Height, cm	163 ± 9	165 ± 10	0.203
Weight, kg	92 ± 20	90 ± 19	0.508
BMI, kg/m^2^	35 ± 7	33 ± 7	0.143
Heart rate, bpm	68 [59–82]	67 [59–78]	0.524
SBP, mmHg	147 ± 27	143 ± 23	0.426
DBP, mmHg	73 [62–80]	75 [68–83]	0.072
History, n (%)			
Hypertension	69 (92)	54 (89)	0.493
Hyperlipidaemia	40 (53)	27 (44)	0.293
Diabetes	46 (61)	22 (36)	**0.003**
IHD	19 (25)	17 (28)	0.739
Smoking	38 (51)	34 (56)	0.556
COPD	9 (12)	9 (15)	0.637
Previous HF hospitalisation	52 (69)	38 (62)	0.388
Medications, n (%)			
ACEi	46 (61)	29 (48)	0.108
ARB	23 (31)	19 (31)	0.952
Beta blocker	45 (60)	47 (77)	**0.035**
Dihydropyridine CCB	25 (33)	14 (23)	0.183
Non-dihydropyridine CCB	2 (3)	4 (7)	0.272
Digoxin	0 (0)	11 (18)	**< 0.001**
Amiodarone	0 (0)	2 (3)	0.114
Diuretic	59 (79)	50 (82)	0.631
Statin	50 (67)	36 (59)	0.357
Metformin	22 (29)	12 (20)	0.196
Sulphonylurea	6 (8)	5 (8)	0.967
Insulin	17 (23)	6 (10)	**0.047**
eGFR, mL/min/1.73m^2^	60 [46–83]	69 [56–83]	0.079
HbA1c, %	6.4 [5.8–7.4]	6.0 [5.7–6.8]	0.378
BNP, ng/L	111 [43–249]	159 [92–271]	0.044
Functional Status			
NYHA Class I/II, n (%)	50 (67)	44 (72)	0.493
NYHA Class III/IV, n (%)	25 (33)	17 (28)	0.493
6MWT distance, m	192 ± 91	200 ± 93	0.615
Minnesota living with HF score	48 ± 24	43 ± 22	0.214
HFA-PEFF score			0.607
0–1	5 (7)	7 (12)	
2–4	40 (53)	30 (49)	
≥ 5	30 (40)	24 (39)	

Data are presented as mean ± standard deviation, median [interquartile range] or number (%) as appropriate. Abbreviations: *BMI* body mass index; *SBP* systolic blood pressure; *DBP* diastolic blood pressure; *eGFR* estimated glomerular filtration rate; *IHD* ischaemic heart disease; *ACEi* angiotensin converting enzyme inhibitor; *ARB* angiotensin II receptor blocker; *CCB* calcium channel blocker; *6MWT* six-minute walk test. *P* value< 0.05 considered statistically significant and are highlighted in bold

### Imaging data

Table 2 summarises imaging data. Those with AF had similar E/e’ values but higher E wave velocities and shorter E wave deceleration times.

**Table 2:** Key imaging data in sinus rhythm versus atrial fibrillation groups

	Sinus rhythm	AF	*P* value*
Echocardiography data			
E wave (cm/s)	75 ± 27	92 ± 27	**0.002**
E deceleration time (ms)	259 ± 78	211 ± 60	**< 0.001**
Septal e’ (cm/s)	6.5 ± 4.6	6.6 ± 1.8	0.733
Lateral e’ (cm/s)	7.7 ± 2.2	8.8. ± 2.6	0.104
E:e’ ratio	12.9 ± 4.7	12.8 ± 4.7	0.858
CMR data			
LV EDVi (mL/m^2^)	71 ± 18	75 ± 18	0.378
LV ESVi (mL/m^2^)	23 ± 10	28 ± 10	**0.028**
LV SVi (mL/m^2^)	47 ± 11	47 ± 12	0.550
LV EF (%)	68 ± 8	63 ± 8	**0.002**
LVMi (g/m^2^)	60 ± 13	60 ± 15	0.461
LVM/EDV (g/mL)	0.89 ± 0.20	0.82 ± 0.17	0.503
GLS (%)	14.7 ± 2.4	13.6 ± 2.9	**0.003**
GCS (%)	18.3 ± 2.8	15.7 ± 3.5	**< 0.001**
Longitudinal PEDSR (s^−1^)	0.53 ± 0.17	0.73 ± 0.28	**< 0.001**
Circumferential PEDSR (s^−1^)	0.67 ± 0.23	0.86 ± 0.33	**0.002**
Maximum LAVi (mL/m^2^)	39 ± 15	61 ± 30	**< 0.001**
Minimum LAVi (mL/m^2^)	20 ± 11	44 ± 27	**< 0.001**
LAV/LV EDV	0.56 ± 0.17	0.81 ± 0.38	**< 0.001**
LA EF (%)	51 ± 12	31 ± 15	**< 0.001**
Presence of LGE, n (%)	34 (45)	29 (50)	0.360
Presence of infarct, n (%)	11 (15)	11 (18)	0.382
Presence of non-ischaemic LGE, n (%)	26 (35)	21 (35)	0.555
Native T1 (ms)	1237 ± 63	1227 ± 84	0.879
Extracellular Volume (%)	26.8 ± 4.1	28.4 ± 4.9	0.092
Stress MBF (mL/min/g)	1.74 ± 0.70	1.57 ± 0.63	0.424
Rest MBF (mL/min/g)	1.22 ± 0.45	0.95 ± 0.32	**0.042**
MPR	1.66 ± 0.72	1.72 ± 0.64	0.722

Data are presented as mean ± standard deviation or number (%) as appropriate. Abbreviations: *AF* atrial fibrillation; *LV* left ventricle; *EDVi* indexed end-diastolic volume; *ESVi* indexed end-systolic volume; *SVi* indexed stroke volume; *EF* ejection fraction; *LVMi* indexed left ventricular mass; *LAVi* indexed left atrial volume; *GCS* global circumferential strain; *GLS* global longitudinal strain; *PEDSR* peak early diastolic strain rate; *LGE* late gadolinium enhancement; *MBF* myocardial blood flow; *MPR* myocardial perfusion reserve. *ANCOVA with age, sex, ethnicity, BMI, diabetes status, systolic BP and eGFR as covariates. *P* value< 0.05 considered statistically significant and are highlighted in bold.

**Fig. 2:**
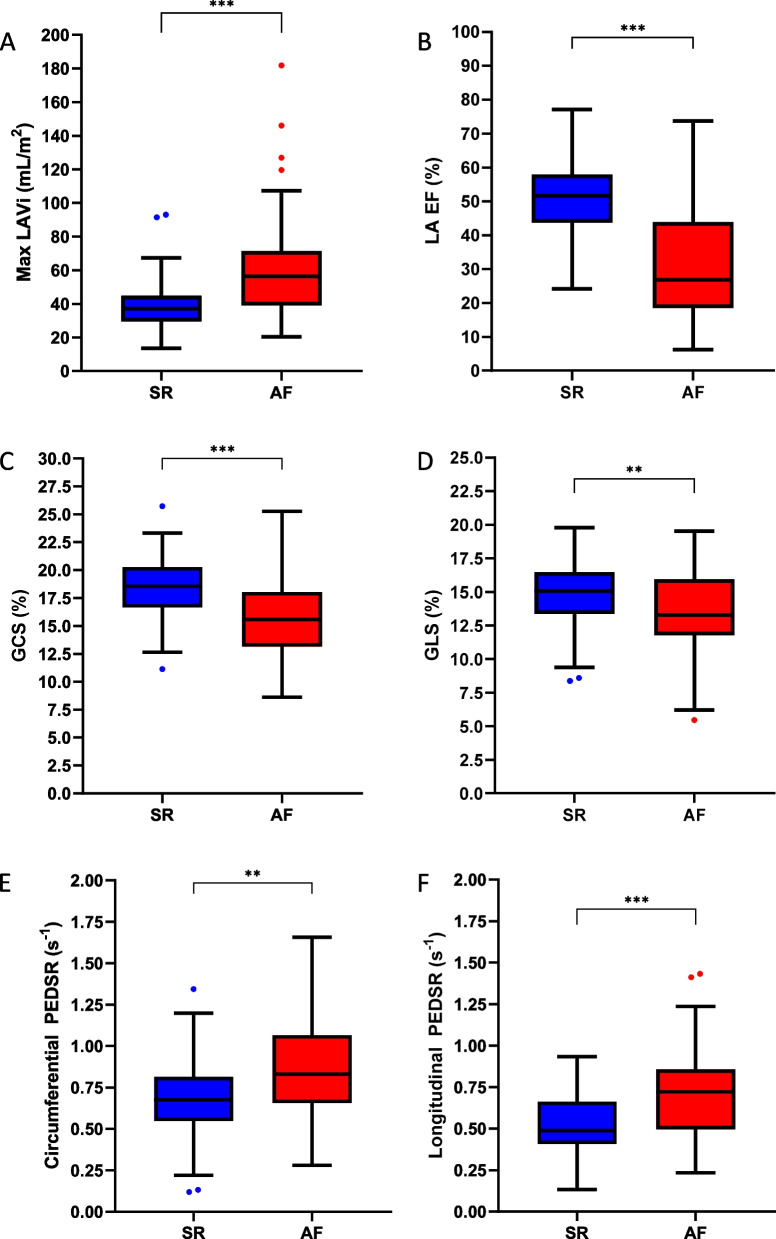
Differences in CMR-derived left atrial measurements (**A**, **B**), systolic strain (**C**,**D**) and diastolic strain rates (**E**–**F**) between the sinus rhythm and AF group. Max LAVi = maximum indexed left atrial volume; LA EF = left atrial ejection fraction; GCS = global circumferential strain; GLS = global longitudinal strain; PEDSR = peak early diastolic strain rate. Significance levels: ** = *p* < 0.01, *** = *p* < 0.001

On CMR, the two groups had similar LV end-diastolic volumes, but the AF group had higher LV end-systolic volumes and lower LV EF in comparison to the SR group. LV mass and LV mass:volume ratios were similar across the groups. Indexed maximum and minimum LA volumes were higher in the AF group and LA EF was lower. GCS and GLS were lower in the AF group. Contrastingly, circumferential and longitudinal PEDSR were higher in the AF group (Fig. 2). Ninety-six participants had T1 mapping performed. No overall differences in focal or diffuse fibrosis were observed between the groups. Quantitative perfusion analysis was undertaken in 99 participants (AF = 43, SR = 56). Despite similar heart rates, the AF group had lower resting but similar stress myocardial blood flow and similar myocardial perfusion reserve compared to the SR group. Right ventricular volumes and EF were similar across the two groups (Supplementary Table S2). Sensitivity analysis excluding participants with paroxysmal AF (21 excluded) demonstrated consistent findings except the AF group had greater diffuse fibrosis compared to the SR group (Supplementary Table S4).

Intra-observer variability analysis demonstrated excellent reliability in both groups for strain measurements (Supplementary Table S6).

### Plasma biomarker analysis

**Fig. 3:**
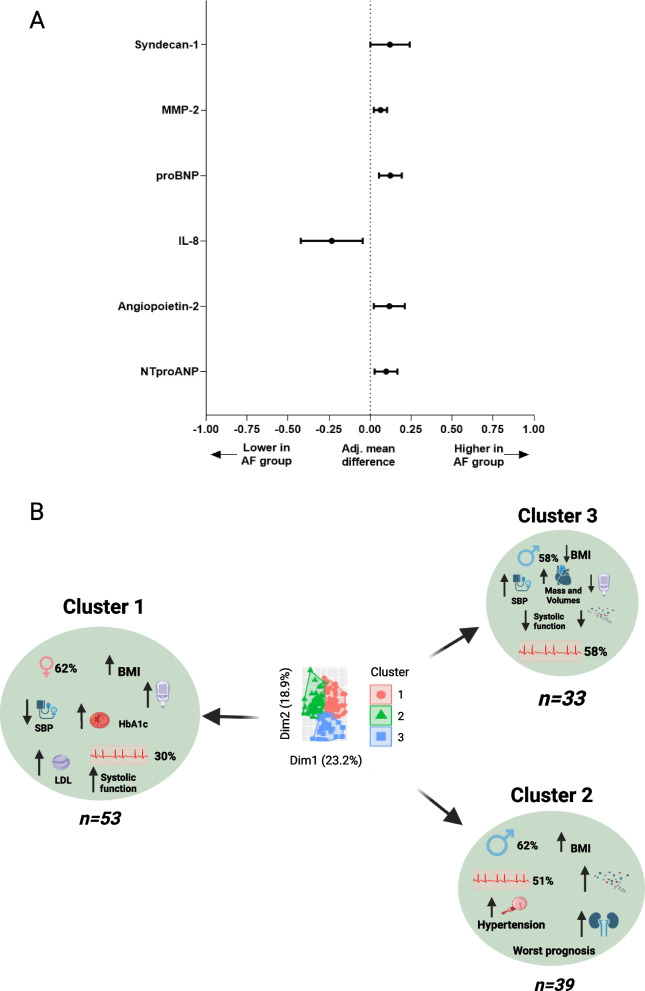
Forest plot (**A**) showing adjusted mean differences in key plasma biomarkers between AF and SR groups. All values are Log10 transformed. Means were adjusted for age, sex, ethnicity, body mass index, diabetes status, systolic blood pressure and estimated glomerular filtration rate. Cluster analysis (**B**) showed three distinct cohorts with cluster 1 had 30% with AF and was made up of mainly females with high BMI, higher prevalence of diabetes with poorer glycaemic control and higher circulating lipids and lower systolic BP and better systolic function. In Cluster 2, 51% had AF and there were more males with higher BMI, an adverse fibroinflammatory profile, poorer kidney function and hypertensive. Cluster 3 had the highest proportion of AF and was mainly male with lower BMI, low prevalence of diabetes, comparatively better fibroinflammatory profile, with increased systolic blood pressure, higher LV mass and biventricular volumes and poorer LV systolic function. Abbreviations: MMP-2 = matrix metalloproteinase-2; BNP = B-type natriuretic peptide; ANP = atrial natriuretic peptide; IL-8 = interleukin-8. Image created using Biorender.com

Circulating levels of NT-proANP (7705 ± 2701 vs. 5749 ± 2764 pg/mL), proBNP (2.25 ± 1.15 vs. 1.68 ± 0.98 pg/mL), angiopoietin-2 (2803 ± 1428 vs. 2048 ± 973 pg/mL), MMP-2 (81,049 ± 19,689 vs. 69,080 ± 18,387 pg/mL) and syndecan-1 (624 ± 410 vs. 559 ± 233 pg/mL) were significantly higher in the AF group compared to the SR group, whereas interleukin-8 levels were lower in the AF group (3.47 ± 2.85 vs. 4.60 ± 3.79 pg/mL) (Fig. 3A). There was a greater proportion of patients in the AF group with high pentraxin-3 level compared to the SR group (32 vs. 16%, *p* = 0.029). Sensitivity analysis excluding participants with paroxysmal AF (21 excluded) showed similar changes in biomarkers except differences in interleukin-8 no longer reached statistical significance (Supplementary Table S4).

### Cluster analysis

The K-means clustering that produced the optimal number of clusters was K = 3 (Fig. 3B). Feature selection identified 25 variables from the dataset that were most important for data partitioning (cluster assignment) and included: BMI, 13 fibro-inflammatory biomarkers and 11 measures of cardiovascular structure and function. The mean values for these variables across the three clusters are provided in Supplementary Table S5 in addition to key baseline characteristics. Cluster assignment was associated with AF status (*p* = 0.023) with Cluster 3 having the greatest proportion with AF (58%) and Cluster 1 (30%) representing the lowest proportion. Age and ethnic breakdown were comparable across the three clusters however, Cluster 1 had the highest proportion of females. The key characteristics by cluster are provided in Fig. 3B. Cluster 1 is a SR predominant HFpEF group characterised by obese females, a higher prevalence of diabetes with poorer glycaemic control and circulating lipids with lower SBP. Cluster 3 with AF predominance is characterised by male sex, lower BMI (within the over-weight category), higher biventricular volumes and greater LV mass with the lowest systolic function and further characterised by the lowest proportion of diabetes, corresponding HbA1c levels and more favourable fibro-inflammatory profile, whilst demonstrating a higher SBP compared to Clusters 1 and 2. Cluster 2 had almost equal AF:SR assignment and is an obese HFpEF group with the most adverse fibro-inflammatory profile, greater proportion of diabetes and highest proportion of hypertension at 100%.

### Clinical outcomes

**Fig. 4:**
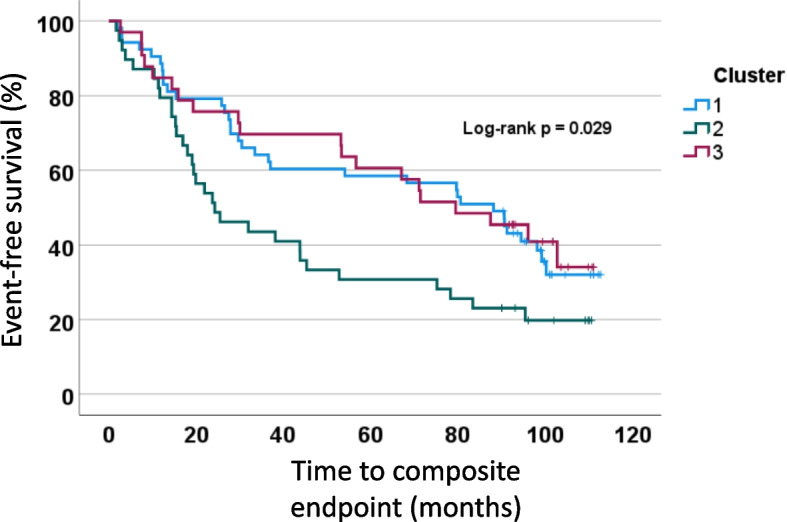
Kaplan-Meier plot showing rate of composite outcomes in the three clusters

Over a median follow-up of 8.5 years, 94 (69%) of participants reached the composite endpoint comprising 57 HF hospitalisations (AF = 27, SR = 30) and 37 deaths (AF = 18, SR = 19). Survival analysis demonstrated no significant difference between the two groups (Log-rank *p* = 0.451) and the presence of AF was not associated with a significant difference in outcome when entered into a Cox regression analysis adjusted for key clinical characteristics (*p* = 0.439). When stratified by cluster, Cluster 1 had 19 HF hospitalisation and 15 deaths, Cluster 2 had 18 HF hospitalisations and 13 deaths, and Cluster 3 had 12 HF hospitalisations and 8 deaths. Kaplan-Meier survival curves for the three clusters are shown in Fig. 4, with cluster 2 having significantly worse outcomes (Log-rank *p* = 0.029).

## Discussion

**Fig. 5:**
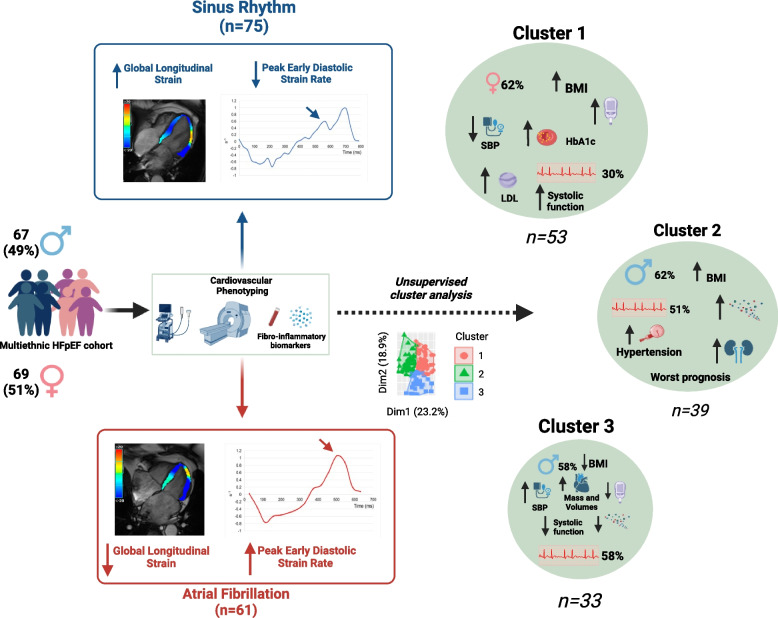
Summary of the study and key findings. In this prospective observational study, adults with HFpEF underwent comprehensive phenotyping with echocardiography, stress perfusion CMR and plasma fibroinflammatory biomarkers. Patients with AF had worse systolic function but higher peak early diastolic strain rate, together with an altered expression of several fibro-inflammatory biomarkers. Cluster analysis demonstrated distinct phenotypes within HFpEF. Image created using Biorender.com

In this detailed multi-modality multidimensional phenotyping study of participants with symptomatic HFpEF, we found that patients with AF have a divergent pattern of myocardial systolic and diastolic mechanics to those in SR and significant differences in several cardiovascular biomarkers. As expected, patients with AF had larger LA volumes and lower LA EF. We demonstrate lower LV systolic function (GCS and GLS) and, for the first time in the literature, have shown enhanced diastolic relaxation (PEDSR) in this group. Clinical outcomes between the two groups were not significantly different suggesting AF status is not the main driver for prognosis in this HFpEF cohort. Cluster analysis identified three distinct groups suggesting phenotypes within this HFpEF cohort. Moreover, Cluster 2 had poorer outcomes, with this group being characterised by male sex, obesity, adverse fibroinflammatory marker profile, diabetes and hypertension. A summary of our findings is shown in Fig. 5.

### Myocardial systolic and diastolic mechanics

Systolic strain measurements such as GLS and GCS are more reflective of myocardial mechanics compared to LV EF, with GLS predominately representing the function of the subendocardial myofibres and GCS representing function of the mid-myocardial myofibres [28]. These measurements are more subtle markers of systolic dysfunction and can be abnormal in HF patients despite a preserved LV EF [29]. Although the AF group in this study did have a marginally lower LV EF compared to the SR group, it remained within normal limits (63 ± 8%). Despite this, we found a lower GLS and GCS in the AF group with values much below those of healthy volunteers [30–32], with average GLS and GCS measuring 16 and 19% respectively in healthy volunteer data from our group using similar methodology [33]. This may suggest a pathological role of impairment of systolic function both in the subendocardial and circumferential myofibres in the development of HFpEF in patients with AF.

PEDSR is a sensitive marker of diastolic dysfunction [34], corresponding to the speed of early LV relaxation and diastolic filling. It is less pressure, cardiac motion and angle dependent than echocardiography-derived E wave velocity, which is dependent on the pressure gradient between the LA and LV [23, 35]. Surprisingly, mean circumferential and longitudinal PEDSR were 28 and 38% higher in the AF group respectively, suggesting an enhanced rate of relaxation of the LV myocardium in early diastole, which is a novel finding. These results are not explained by differences in age given our ANCOVA included age as a covariate. Furthermore, PEDSR declines with age, and our AF group were older than the SR group [36]. Traditionally, it was thought patients with systolic dysfunction tend to also have diastolic dysfunction, with worsening diastolic dysfunction leading to progressive longitudinal systolic dysfunction [37]. However, our data are contrary to this. PEDSR is a measure of diastolic function of the LV, therefore a higher PEDSR may reflect an adaptive suction response to a poorer LA function which is represented by the reduced LA EF found in our patients with AF. As PEDSR corresponds to the early filling of the LV rather than late filling, the missing atrial ‘kick’ in AF should instead impact late diastole.

### Systemic fibro-inflammation

Our data have also demonstrated significant differences in key plasma biomarkers which may, together with the changes described thus far, suggest a unique pathophysiological mechanism of HFpEF in patients with AF. For example, levels of MMP-2 were higher in the AF patients, a finding that has been shown in other studies [8, 38]. MMPs play a key role in structural remodelling of the LA in AF patients and degrade components of the extracellular matrix leading to cardiac fibrosis, which is a key process in the development and progression of HF [39, 40]. Furthermore, intracellularly MMP-2 is able to target important components of the cardiac sarcomere such as troponin I, actin, myosin, titin and α-actinin, potentially impacting directly on systolic and diastolic function [41]. In myocardial ischaemia, MMP-2 has been shown to localise at the sarcomere Z disc region and its proteolytic activity leads to degradation of titin [42]. Further work is needed to evaluate the relative impact of MMP-2 on different sarcomeric proteins in AF.

In keeping with our findings, Angiopoeitin-2 has also been associated with AF in other studies [43] and has been associated with adverse cardiac remodelling [44] as well as both systolic and diastolic dysfunction [45], albeit in non-AF patients. Angiopoeitin-2 plays an important role in angiogenesis and delivers its impact on vascular structure and function via vascular endothelial growth factor [44], potentially leading to arterial stiffness [46], which is known to be associated with HFpEF [47].

The acute phase inflammatory glycoprotein, pentraxin-3, was elevated in our AF group which has been previously reported in other cohorts [48] and has been purported to have a prognostic role in HF [49].

### Cluster analysis

The unsupervised K-means clustering identified three separate clusters, each with a different clinical, imaging and fibroinflammatory profile. This suggests that within our cohort of HFpEF there are distinct phenotypes but these are not purely driven by AF status. Whilst other groups have used unsupervised cluster analysis in larger HFpEF cohorts, [50–52] and some have also identified 3 clusters, their data lack the deep phenotyping that our data offer including multi-modality imaging with TTE and CMR together with a detailed fibroinflammatory marker profile. In our study, the clustering was designed, a priori, based on key clinical, imaging and fibroinflammatory variables with feature selection employed to determine the specific sub-set of these that drives cluster assignment. The clusters were defined by echocardiographic measures of diastolic function, CMR measures of cardiac structure and function, and biomarkers covering inflammatory and fibrotic pathways. This is partly in line with the study by Segar et al., [52] who also found LV systolic and diastolic function (echocardiography measured LV EF and E/a ratio) were discriminatory variables between groups.

In our study, cluster assignment also involved key fibroinflammatory biomarkers that are known to be associated with cardiovascular disease (Supplementary Table S3). Beyond MMP-2 and angiopoietin-2 described above, this included: adiponectin, an anti-inflammatory hormone which has been shown to be independently associated with the development of AF [53] and predictive of mortality in the HF population [54]; Fatty acid binding protein-4 which has also been associated with cardiac function and remodelling [55]; and renin, which is known to play a crucial role in the renin-angiotensin-aldosterone system, and is associated with cardiac remodelling [56]. Unsurprisingly, NTproANP and proBNP, which are released as a response to cardiac wall tension and represent key biomarkers for HF, were also important discriminatory variables in clustering and were shown to be highest in Cluster 2 with the worst prognosis.

### Clinical outcomes

During a relatively long follow-up (median 8.5 years), when comparing only AF with SR, there was no significant difference with respect to the composite endpoint (HF hospitalisation or all-cause death). This is contrary to other studies which have found AF to be associated with worse cardiovascular outcomes [6, 7]. This discrepancy could be attributed to differences in our study cohort compared to some of these registry-based data. For example, the Swedish HF registry data had a larger sample size (*n* = 9595 with HFpEF) to demonstrate a small but significant difference in hazard ratio (HR = 1.11). In comparison, our study involves a smaller sample size from a single centre and was not powered to demonstrate differences in outcomes between AF and SR specifically. Additionally, the AF group in our cohort were less symptomatic with lower proportions of patients with NYHA class III/IV symptoms (28%) compared to the ESC HF long term registry (60%) and the Swedish HF registry (42%). Patient selection may also play a role as our cohort was made up of patients with a clinical diagnosis of HF who chose to take part in a prospectively recruited study in comparison to the non-selective nature of registry data. Interestingly in some studies, the poorer outcome in AF patients is no longer seen once adjusted for important covariates [57], suggesting the different pathophysiology seen in our study likely accounts for only a small average effect on prognosis. This is in line with the outcomes observed for our three phenotypes with Cluster 2, which has the more adverse fibroinflammatory profile, having worse outcomes over Clusters 1 and 3. Given that the key cardiac structural (biventricular volumes and LV mass) and functional (LV systolic function) parameters were not worse in Cluster 2, we postulate that the poorer outcomes may be driven by this elevated fibroinflammatory state.

### Strengths and limitations

Key strengths to this study include it being one of the largest CMR studies in a multi-ethnic HFpEF population of equal sex distribution who underwent extensive non-invasive phenotyping using echocardiography, stress perfusion CMR, functional capacity assessment and plasma biomarkers. A major strength is the standardisation of our imaging approaches in comparison to other non-prospective studies in the HFpEF population. The cluster analysis was unsupervised with cluster number driven by the data and thus reducing bias.

There are some important limitations. Firstly, the sample size in this group is modest, which may have limited further between-group differences being observed. There were some differences in baseline characteristics between the AF and SR groups as discussed in the results section which acts as a limitation, but we did adjust for these covariates during the comparison of our group results. Further, the study was not specifically powered to assess differences in outcomes between patients with AF and those in SR. Only 96 participants had parametric mapping performed which may explain the lack of a statistically significant difference in extracellular volume fraction in our study compared to others [14]. AF is a heterogenous condition and the core analysis did not characterise participants into different types of AF and they did not have an implantable loop recorder as part of this observational study. We therefore were also unable to monitor change in AF status during follow up or assess the impact of AF burden. However, sensitivity analysis was performed excluding those with paroxysmal AF which showed largely consistent findings.

It’s important to address the accuracy of strain measurements in patients with AF. At our centre, we routinely use prospective ECG-gating in patients with AF. Images are constructed over multiple heart beats and therefore may be impacted by the presence of AF. Prospectively-gated images miss the final part of diastole which could underestimate LV volumes and systolic strain but this should not affect PEDSR, which occurs early in diastole. Both retrospective and prospective imaging are acquired using a repetition time of 3.1 ms. Retrospective cine imaging is then reconstructed to 30 phases providing a temporal resolution of 35 ms, whereas prospective cine imaging provides a temporal resolution of 35-46 ms which may affect the measurement of PEDSR. Despite the differences, intra-observer variability was excellent in both groups. The number of plasma fibroinflammatory biomarkers assessed were relatively small (49 biomarkers) and therefore further important differences may be present in this population but not detected in this study. Finally, the data presented here are a retrospective analysis of prospectively collected data in a study for which the primary aim was not to assess the differences in patients with AF compared to SR. Collectively the data should, therefore, be viewed as exploratory and hypothesis-generating.

## Conclusions

In HFpEF patients, we have shown that AF is associated with lower LV systolic function but higher LV diastolic strain rate compared to patients in SR. Our cluster analysis suggests distinct phenotypes within HFpEF which extend beyond simply AF status. The identified clusters, associated clinical characteristics and outcomes require validation in larger longitudinal studies.

### Supplementary Information

**Additional file 1.**

## Data Availability

The datasets used and/or analysed during the current study are available from the corresponding author on reasonable request.

## Abbreviations

6MWT: Six-minute walk test
AF: Atrial fibrillation
BMI: Body mass index
BNP: B-type natriuretic peptide
CMR: Cardiovascular magnetic resonance
ECG: Electrocardiogram
eGFR: Estimated glomerular filtration rate
GCS: Global circumferential strain
GLS: Global longitudinal strain
HFpEF: Heart failure with preserved ejection fraction
LA: Left atrium
LGE: Late gadolinium enhancement
LV: Left ventricle
MMP-2: Matrix metalloproteinase-2
NYHA: New York heart association
NT-proANP: N-terminal proatrial natriuretic peptide
PEDSR: Peak early diastolic strain rate
SBP: Systolic blood pressure
SR: Sinus rhythm
TTE: Transthoracic echocardiography
