# Supplementary material for: Is atrial fibrillation in HFpEF a distinct phenotype? Insights from multiparametric MRI and circulating biomarkers
Source: BMC Cardiovasc Disord. 2024 Feb 7;24:94. doi: 10.1186/s12872-024-03734-0 (PMC10848361; doi:10.1186/s12872-024-03734-0)
Supplement: Supplementary file 1 — Additional file 1. [file 12872_2024_3734_MOESM1_ESM.docx]

# **Supplemental material**

## Plasma biomarkers

A number of biomarkers had multiple values reported at the lower-limit of detection distorting data distribution. To reduce this any biomarkers with >80% data at the lower detection limit were excluded. Then, if >10% of the data were at the lower detection limit that biomarker was dichotomised. The threshold for dichotomising calculated by removing the values at lower detection limit and calculating the mean of the Log10 of the remaining values (values below the mean were classed as “0 = low levels” and values above the mean were classed as “1=high levels”). All other biomarker data were reviewed and depending on data distribution either remained as raw values or log10 transformed.

Table S1: List of biomarkers assessed

| Adiponectin | Interleukin-1beta | proBNP |
| --- | --- | --- |
| Angiopoietin-2 | Interleukin-6 | P-selectin |
| Chitinase-3-Like-1 | Interleukin-8 | Renin |
| C-reactive protein | Kidney injury molecule-1 | Serpin-E1-PAI1 |
| Cystatin-C | Matrix metalloproteinase-12 | Suppression of tumorigenicity-2 |
| Endoglin CD105 | Matrix metalloproteinase -2 | Syndecan-1 |
| Endostatin | Matrix metalloproteinase -3 | Syndecan-4 |
| Endothelin-1 | Matrix metalloproteinase -7 | Tenascin C |
| Fatty acid binding protein-1 | Matrix metalloproteinase -8 | Tissue inhibitor of metalloproteinases-1 |
| Fatty acid binding protein-4 | Matrix metalloproteinase -9 | Tissue inhibitor of metalloproteinases-4 |
| Fas | Myeloperoxidase | Tumour necrosis factor-receptor 1 |
| Fibroblast growth factor-23 | Neutrophil gelatinase-associated lipocalin | Tumour necrosis factor-alpha |
| Fibroblast growth factor-21 | NTProANP | Tumour necrosis factor-receptor- II |
| Galectin-3 | Osteoprotegerin | Troponin T |
| Growth differentiation factor-15 | Osteopontin | Vascular endothelial growth factor |
| Intercellular adhesion molecule-1 | Pentraxin 3 | Vascular endothelial growth factor-receptor1 |
| Interleukin-10 |  |  |

Table S2: CMR results of the right ventricle of HFpEF participants with and without AF.

|  | Sinus rhythm | AF | P value* |
| --- | --- | --- | --- |
| RV EDVi (mL/m^2^) | 77 ± 17 | 83 ± 21 | 0.607 |
| RV ESVi (mL/m^2^) | 35 ± 13 | 40 ± 15 | 0.366 |
| RV SVi (mL/m^2^) | 43 ± 9 | 43 ± 11 | 0.753 |
| RV EF (%) | 56 ± 8 | 52 ± 10 | 0.147 |

Data are presented as mean ± standard deviation. Abbreviations: RV = right ventricle; EDVi = indexed end-diastolic volume; ESVi = indexed end-systolic volume; SVi = indexed stroke volume; EF = ejection fraction. *ANCOVA with age, sex, ethnicity, BMI, diabetes status, systolic BP and eGFR as covariates

| Clinical | Imaging | Plasma biomarkers |
| --- | --- | --- |
| Age | E wave | Cystatin-C |
| BMI | E deceleration time | Matrix metalloproteinase-12 |
| eGFR | E:e’ ratio | NTproANP |
| SBP | LV EDVi | Tissue inhibitor of metalloproteinases-4 |
|  | LV ESVi | Log10 Adiponectin |
|  | LV SVi | Log10 Angiopoietin-2 |
|  | LV EF | Log10 BNP |
|  | LVMi | Log10 Chitinase-3-Like-1 |
|  | LVM/EDV | Log10 C-reactive protein |
|  | GCS | Log10 Endoglin |
|  | GLS | Log10 Endostatin |
|  | Longitudinal PEDSR | Log10 Fatty acid binding protein-4 |
|  | Circumferential PEDSR | Log10 Fas |
|  | Maximum LAVi | Log10 Fibroblast growth factor-23 |
|  | LA EF | Log10 Galectin-3 |
|  | RV EDVi | Log10 Growth differentiation factor-15 |
|  | RV ESVi | Log10 Intercellular adhesion molecule-1 |
|  | RV SVi | Log10 Interleukin-6 |
|  | RV EF | Log10 Interleukin-8 |
|  |  | Log10 Matrix metalloproteinase-2 |
|  |  | Log10 Matrix metalloproteinase-3 |
|  |  | Log10 Matrix metalloproteinase-7 |
|  |  | Log10 Matrix metalloproteinase-8 |
|  |  | Log10 Matrix metalloproteinase-9 |
|  |  | Log10 Myeloperoxidase |
|  |  | Log10 Neutrophil gelatinase-associated lipocalin |
|  |  | Log10 Osteoprotegerin |
|  |  | Log10 Osteopontin |
|  |  | Log10 proBNP |
|  |  | Log10 P-selectin |
|  |  | Log10 Renin |
|  |  | Log10 Serpin-E1-PAI1 |
|  |  | Log10 Suppression of tumorigenicity-2 |
|  |  | Log10 Syndecan-1 |
|  |  | Log10 Syndecan-4 |
|  |  | Log10 Tenascin-C |
|  |  | Log10 Tissue inhibitor of metalloproteinases-1 |
|  |  | Log10 Tumour necrosis factor-receptor 1 |
|  |  | Log10 Vascular endothelial growth factor |
|  |  | Log10 Vascular endothelial growth factor-receptor 1 |

Table S3: List of variables used for cluster analysis

Abbreviations: BMI = body mass index; eGFR = estimated glomerular filtration rate; SBP = systolic blood pressure; DBP = diastolic blood pressure; 6MWT = six minute walk test; HF = heart failure; LV = left ventricle; EDVi = indexed end-diastolic volume; ESVi = indexed end-systolic volume; SVi = indexed stroke volume; EF = ejection fraction; GCS = global circumferential strain; GLS = global longitudinal strain; PEDSR = peak early diastolic strain rate; LAVi = indexed left atrial volume; RV = right ventricle.

|  | Sinus rhythm  (n=75) | AF  (n=40) | P value* |
| --- | --- | --- | --- |
| CMR data | | | |
| LV EDVi (mL/m^2^) | 71 ± 18 | 72 ± 19 | 0.869 |
| LV ESVi (mL/m^2^) | 23 ± 10 | 27 ± 9 | 0.071 |
| LV SVi (mL/m^2^) | 47 ± 11 | 45 ± 12 | 0.208 |
| LV EF (%) | 68 ± 8 | 62 ± 7 | **0.001** |
| LVMi (g/m^2^) | 60 ± 13 | 60 ± 17 | 0.467 |
| LVM/EDV (g/mL) | 0.89 ± 0.20 | 0.86 ± 0.18 | 0.808 |
| GLS (%) | 14.7 ± 2.4 | 12.9 ± 2.9 | **<0.001** |
| Longitudinal PEDSR (s^-1^) | 0.53 ± 0.17 | 0.83 ± 0.26 | **<0.001** |
| Maximum LAVi (mL/m^2^) | 39 ± 15 | 70 ± 31 | **<0.001** |
| Minimum LAVi (mL/m^2^) | 20 ± 11 | 55 ± 26 | **<0.001** |
| LAV/LV EDV | 0.56 ± 0.17 | 0.96 ± 0.14 | **<0.001** |
| LA EF (%) | 51 ± 12 | 22 ± 9 | **<0.001** |
| Presence of LGE, n (%)  Presence of infarct, n (%)  Presence of non-ischaemic LGE, n (%) | 34 (45)  11 (15)  26 (35) | 18 (45)  8 (20)  12 (30) | 0.973  0.463  0.612 |
| Native T1 (ms) | 1237 ± 63 | 1231 ± 87 | 0.630 |
| Extracellular Volume (%) | 26.8 ± 4.1 | 29.6 ± 5.0 | **0.011** |
| Stress MBF (mL/min/g) | 1.74 ± 0.70 | 1.46 ± 0.67 | 0.204 |
| Rest MBF (mL/min/g) | 1.22 ± 0.45 | 0.89 ± 0.32 | **0.026** |
| MPR | 1.66 ± 0.72 | 1.68 ± 0.78 | 0.873 |
| Biomarker data | | | |
| NTproANP | 3.71 ± 0.23 | 3.89 ± 0.20 | **<0.001** |
| proBNP | 0.18 ± 0.17 | 0.38 ± 0.18 | **<0.001** |
| Angiopoietin-2 | 3.27 ± 0.20 | 3.42 ± 0.29 | **0.003** |
| Matrix metalloproteinase-2 | 4.83 ± 0.11 | 4.91 ± 0.12 | **<0.001** |
| Syndecan-1 | 2.68 ± 0.37 | 2.77 ± 0.21 | **0.033** |
| Interleukin-8 | 0.57 ± 0.27 | 0.40 ± 0.58 | 0.110 |
| Pentraxin-3, n (%) High | 11 (16%) | 15 (40%) | **0.006** |

Table S4: Key imaging and biomarker data in the sinus rhythm and AF groups with exclusion of participants with paroxysmal AF.

Data are presented as mean ± standard deviation or number (%) as appropriate. Abbreviations: AF = atrial fibrillation; LV = left ventricle; EDVi = indexed end-diastolic volume; ESVi = indexed end-systolic volume; SVi = indexed stroke volume; EF = ejection fraction; LVMi = indexed left ventricular mass; LAVi = indexed left atrial volume; GCS = global circumferential strain; GLS = global longitudinal strain; PEDSR = peak early diastolic strain rate; LGE = late gadolinium enhancement; MBF = myocardial blood flow; MPR = myocardial perfusion reserve. *Adjusted for age, sex, ethnicity, BMI, diabetes status, systolic BP and eGFR as covariates. P value<0.05 considered statistically significant.

|  | Cluster 1  (n=53) | Cluster 2  (n=39) | Cluster 3  (n=33) | P value |
| --- | --- | --- | --- | --- |
| Clinical | | | |  |
| Age | 70.81 ± 8.26 | 74.15 ± 9.66 | 73.09 ± 11.10 | 0.210 |
| Sex, n (%) female | 33 (62) | 15 (38) | 14 (42) | **0.049** |
| SBP | 140 ± 22 | 146 ± 29 | 154 ± 25 | **0.016** |
| eGFR | 68 ± 21 | 51 ± 15 | 82 ± 13 | **0.021** |
| BMI (kg/m^2^) * | 36 ± 7 | 33 ± 7 | 30 ± 6 | **<0.0001** |
| HbA1c (%) | 6.69 ± 1.02 | 6.45 ± 1.05 | 6.34 ± 1.54 | 0.180 |
| AF, n (%) | 16 (30) | 20 (51) | 19 (58) | **0.023** |
| Diabetes, n (%) | 31 (58) | 21 (54) | 10 (30) | **0.032** |
| Hypertension | 46 (87) | 39 (100) | 28 (85) | **0.022** |
| Key imaging variable | | | |  |
| E wave (cm/s)* | 73 ± 22 | 101 ± 29 | 76 ± 27 | **<0.0001** |
| E:e’ ratio* | 12 ± 4 | 16 ± 5 | 11 ± 4 | 0.820 |
| LV EDVi (mL/m^2^)* | 60 ± 11 | 79 ± 16 | 90 ± 15 | **<0.0001** |
| LV ESVi (mL/m^2^)* | 19 ± 7 | 28 ± 8 | 36 ± 11 | **<0.0001** |
| LV SVi (mL/m^2^)* | 41 ± 9 | 52 ± 12 | 54 ± 8 | **<0.0001** |
| LV EF (%)* | 69 ± 8 | 65 ± 8 | 61 ± 7 | **<0.0001** |
| LVMi (g/m^2^)* | 52 ± 9 | 65 ± 14 | 69 ± 15 | **<0.0001** |
| GCS (%)* | 18.5 ± 2.8 | 16.4 ± 3.7 | 15.9 ± 3.3 | **<0.0001** |
| LA EF (%)* | 48 ± 15 | 36 ± 16 | 39 ± 19 | **<0.0001** |
| RV EDVi (mL/m^2^)* | 69 ± 14 | 86 ± 15 | 96 ± 20 | **<0.0001** |
| RV ESVi (mL/m^2^)* | 31 ± 11 | 39 ± 12 | 46 ± 16 | **<0.0001** |
| Key plasma biomarkers | | | |  |
| Cystatin-C (pg/mL)* | 770778 ± 146412 | 1046200 ± 174851 | 673816 ± 119381 | 0.100 |
| NTproANP (pg/mL)* | 5157 ± 2105 | 8689 ± 2596 | 6819 ± 2686 | **<0.0001** |
| Log10 Adiponectin (pg/mL)* | 7.65 ± 0.25 | 7.86 ± 0.24 | 7.85 ± 0.28 | **<0.0001** |
| Log10 Angiopoietin-2 (pg/mL)* | 3.27 ± 0.17 | 3.43 ± 0.23 | 3.30 ± 0.23 | **<0.0001** |
| Log10 Endostatin (pg/mL)* | 5.15 ± 0.15 | 5.38 ± 0.12 | 5.10 ± 0.10 | 0.290 |
| Log10 Fatty acid binding protein-4 (pg/mL)* | 4.03 ± 0.42 | 4.20 ± 0.28 | 3.58 ± 0.73 | **<0.0001** |
| Log10 Galectin-3 (pg/mL)* | 3.85 ± 0.13 | 3.93 ± 0.12 | 3.78 ± 0.10 | **<0.0001** |
| Log10 Matrix metalloproteinase-2 (pg/mL)* | 4.80 ± 0.09 | 4.93 ± 0.10 | 4.87 ± 0.09 | **<0.0001** |
| Log10 Neutrophil gelatinase-associated lipocalin (pg/mL)* | 4.58 ± 0.15 | 4.82 ± 0.11 | 4.55 ± 0.11 | **0.049** |
| Log10 proBNP (pg/mL)* | 0.15 ± 0.13 | 0.35 ± 0.22 | 0.25 ± 0.16 | **<0.0001** |
| Log10 Renin (pg/mL)* | 2.66 ± 0.48 | 2.79 ± 0.38 | 2.38 ± 0.40 | **<0.0001** |
| Log10 Suppression of tumorigenicity-2 (pg/mL)* | 3.75 ± 0.18 | 3.90 ± 0.17 | 3.83 ± 0.20 | **<0.0001** |
| Log10 Tumour necrosis factor-receptor 1 (pg/mL)* | 3.70 ± 0.15 | 3.94 ± 0.13 | 3.59 ± 0.13 | **0.0007** |

Table S5: Average values of key baseline characteristics across the clusters and variables identified to be important for cluster assignment

Data are presented as mean ± standard deviation or number (%) as appropriate. Abbreviations: SBP = systolic blood pressure; eGFR = estimated glomerular filtration rate; BMI = body mass index; AF = atrial fibrillation; LV = left ventricle; EDVi = indexed end-diastolic volume; ESVi = indexed end-systolic volume; SVi = indexed stroke volume; EF = ejection fraction; LVMi = indexed left ventricular mass; GCS = global circumferential strain; RV = right ventricle. * = important for cluster assignment. P value<0.05 considered statistically significant.

Table S6: Intra-observer variability of strain measurements in the SR and AF groups.

Abbreviations: SR = sinus rhythm, AF = atrial fibrillation, GCS = global circumferential strain, GLS = global longitudinal strian, PEDSR = peak early diastolic strain rate.

|  | SR (n=10) | | | | AF (n=10) | | | |
| --- | --- | --- | --- | --- | --- | --- | --- | --- |
|  | **ICC** | **Bias** | **LLOA** | **ULOA** | **ICC** | **Bias** | **LLOA** | **ULOA** |
| GCS | 0.998 | 0.054 | -0.556 | 0.664 | 0.979 | 0.086 | -1.335 | 1.507 |
| GLS | 0.951 | 0.372 | -1.476 | 2.220 | 0.962 | -0.404 | -2.384 | 1.576 |
| Longitudinal PEDSR | 0.952 | -0.016 | -0.129 | 0.097 | 0.949 | -0.066 | -0.311 | 0.178 |
| Circumferential PEDSR | 0.986 | -0.005 | -0.063 | 0.052 | 0.995 | -0.016 | -0.102 | 0.070 |
